# The Scope of Pediatric Forensic Psychiatric Evaluations in Student Run Asylum Clinics

**DOI:** 10.1007/s10903-025-01704-y

**Published:** 2025-05-31

**Authors:** Elizabeth Sun, Alvina Liang, Mae Wimbiscus, Caroline Castleman, Jeffrey Stovall, Elizabeth S Rose

**Affiliations:** 1 Vanderbilt University School of Medicine, Nashville, USA; 2 Vanderbilt University Medical Center, Department of Psychiatry, Nashville, USA; 3 Vanderbilt Institute for Global Health, Vanderbilt University Medical Center, Nashville, USA

**Keywords:** Asylum, Refugee, Forensic medical evaluation, Psychiatry, Medical student

## Abstract

Student-run asylum clinics (SRACs) are organizations that provide *pro bono* forensic medical evaluations to support the legal asylum cases of individuals seeking refuge away from their home country, increasing the likelihood that asylum status is granted. Although thousands of children seek asylum in the U.S. every year, there are fewer forensic medical evaluations performed for children relative to adults. This cross-sectional survey study investigates the extent of forensic medical evaluation services provided to children by SRACs in the United States. An online, cross-sectional survey evaluating the scope of pediatric practice and barriers to completing pediatric forensic evaluations was distributed to 27 student-run asylum clinics. Of fourteen organizations that completed the survey, 35.7% (*n* = 5) received referrals in the past year for pediatric forensic evaluations, while 92.9% (*n* = 13) received referrals for adult evaluations. 53.8% (*n* = 7) of respondents indicated that a barrier to performing pediatric evaluations was a limited number of physicians specialized in this area. There is likely an unmet need for pediatric forensic medical evaluations, and a significant barrier is a shortage of physicians specialized in performing these evaluations. Given the large volume of children who seek asylum in the United States every year, it is imperative to train additional physicians to perform pediatric evaluations to expand asylum services nationwide to children.

## Background

An individual seeking asylum is defined as a refugee who is pursuing protection to remain in the United States [1]. Often, protection is sought due to an inability to return to their country of nationality or residence because of a well-founded fear of persecution based on race, religion, nationality, membership of particular social groups, or political opinion [1]. Among asylum seekers, thousands are children. While children with legal guardians applying for asylum are granted asylum status once their guardians’ asylum applications are approved, children under the age of 18 without lawful immigration status and without legal guardians are considered to be unaccompanied children and file for asylum status independently [2]. The United States Office of Refugee Resettlement, which is responsible for the care and custody of unaccompanied children, received 118,938 referrals for the care of unaccompanied children in 2023 [2]. In 2023, there were 456,750 affirmative asylum case filings. Of those cases, approximately 11,875 were unaccompanied children, an increase from 2022, when approximately 9,892 unaccompanied children filed for asylum [3].

Refugee children, despite being a heterogeneously defined group, are at a greater risk of mental health disorders such as post-traumatic stress disorder, depression, and anxiety compared to children worldwide [4–6]. For individuals seeking asylum, forensic medical and psychiatric evaluations (also known as psychologic evaluations or psychiatric assessments) can increase the likelihood that individuals are granted asylum status from 38 to 89% nationally [7]. Forensic clinical evaluations are focused clinical exams, most commonly in the form a psychiatric interview, that correlates an individual’s history of persecution in their country of origin to their exam findings, which is documented and shared with the individual’s attorney and used to corroborate their legal case [8]. The interview and physical exam (if performed) typically take two to three hours to complete [9] followed by an additional few hours after the evaluation to document findings in the form of a medical affidavit. Most commonly, these evaluations are performed by physicians and mental health specialists within academic institutions that are partnered with human rights organizations such as Physicians for Human Rights [7–9]. Medical and psychiatric asylum evaluations are also provided by private practice clinics, various refugee clinics, and international humanitarian organizations such as Doctors of the World [7]. In the setting of an increasing need for asylum evaluations, student-run asylum clinics (SRACs) emerged as entities (often medical school-affiliated) that provide *pro bono* forensic medical evaluations to individuals seeking asylum in collaboration with their legal representation [10, 11].

Despite the documented utility of forensic evaluation [7, 12] and the high prevalence of children seeking asylum in the United States, there is a paucity of evaluations being performed for minors in comparison to adults [13, 14]. Moreover, literature on the topic of pediatric forensic evaluations is notably sparse in comparison to literature on the topic of adult forensic evaluations. While a couple of SRACs have shared their experiences conducting pediatric forensic evaluations [14, 15], a nationwide survey in 2021 targeting clinicians who work with immigrants identified merely 28 providers who reported performing forensic evaluations for the pediatric populations, half of which were child abuse pediatricians [13].

There have not been prior investigations as to why there is a shortage of pediatric forensic evaluations. Many types of providers are capable of performing pediatric forensic evaluations, including pediatricians, pediatric gynecologists, adolescent medicine physicians, child abuse pediatricians, family physicians, child psychiatrists and psychologists, physician assistants, social workers, and nurse practitioners [4]. However, many of the existing training programs and materials for providers performing forensic medical and psychiatric evaluations are geared towards the adult population rather than the pediatric population [16]. It is essential that providers conducting pediatric forensic evaluations have training that enable them to conduct the evaluation in developmentally appropriate language, assess child developmental stages, understand the nuances of informed consent, and understand how trauma impacts children differently from adults [16, 17]. The lack of pediatric forensic evaluation services could indicate that there is a lack of available training.

Currently, there are at least 44 medical schools with organizations that provide adult forensic evaluations for individuals seeking asylum or additional human rights visas such as U-visas and T-visas; however, little is known regarding the extent to which of these organizations provide pediatric evaluations. The purpose of this study is to investigate the perceived need and extent of forensic medical evaluation services provided to children, whether accompanied or unaccompanied, by SRACs in the context of an increasing number of asylum-seeking children within the United States.

## Methods

An online survey was created to collect information on pediatric forensic evaluation practices in SRACs. Study data were collected and managed using REDCap electronic data capture tools [18, 19]. The REDCap platform is funded by UL1 TR000445 from NCATS/NIH. The survey questions included: (1) the average number of forensic evaluations conducted per month; (2) whether the organization has noticed the need for pediatric forensic evaluations in their area; (3) whether their organization received referrals in the past year for pediatric forensic evaluations; (4) the number of referrals for pediatric evaluations received (meaning the number of evaluation requests received) and (5) completed in the past three years; (6) the number of adult referrals received and (7) completed over the past three years; and (8) barriers encountered when considering or attempting to provide forensic evaluations for pediatric populations.

A list of 44 SRACs was compiled using public online resources including organizational websites published by The Society of Asylum Medicine and Physicians for Human Rights. Student-run organizations affiliated with medical schools or local health systems that provide forensic medical evaluations to support human rights cases were defined as asylum clinics. SRAC contact information for 27 of these organizations was obtained via internet search or through known contacts. Surveys were emailed to contacts in April 2024. Contacts were sent up to three reminders to complete the survey and given up to eight weeks to complete the survey. The Institutional Review Board (IRB) reviewed the study protocol and waived the requirement for informed consent. All study data was stored in secured institutional databases and accessible only to trained study personnel.

Data was reviewed and summarized descriptively. Proportions, means, and standard deviations were calculated using Microsoft Excel. Figures were generating using Microsoft Excel.

## Results

Of the fourteen organizations that completed the survey, 35.7% (*n* = 5) answered “yes” to noticing a need for pediatric forensic evaluations in their area, and 35.7% (*n* = 5) answered “yes” to receiving referrals in the past year for pediatric forensic evaluations. Of these five organizations that received referrals, four specified the number of pediatric forensic evaluation referrals received (mean = 9.3, SD = 13.2, range = 2–29) and completed (mean = 8.5, SD = 13.1, range = 0–28) in the past three years.

**Fig. 1:**
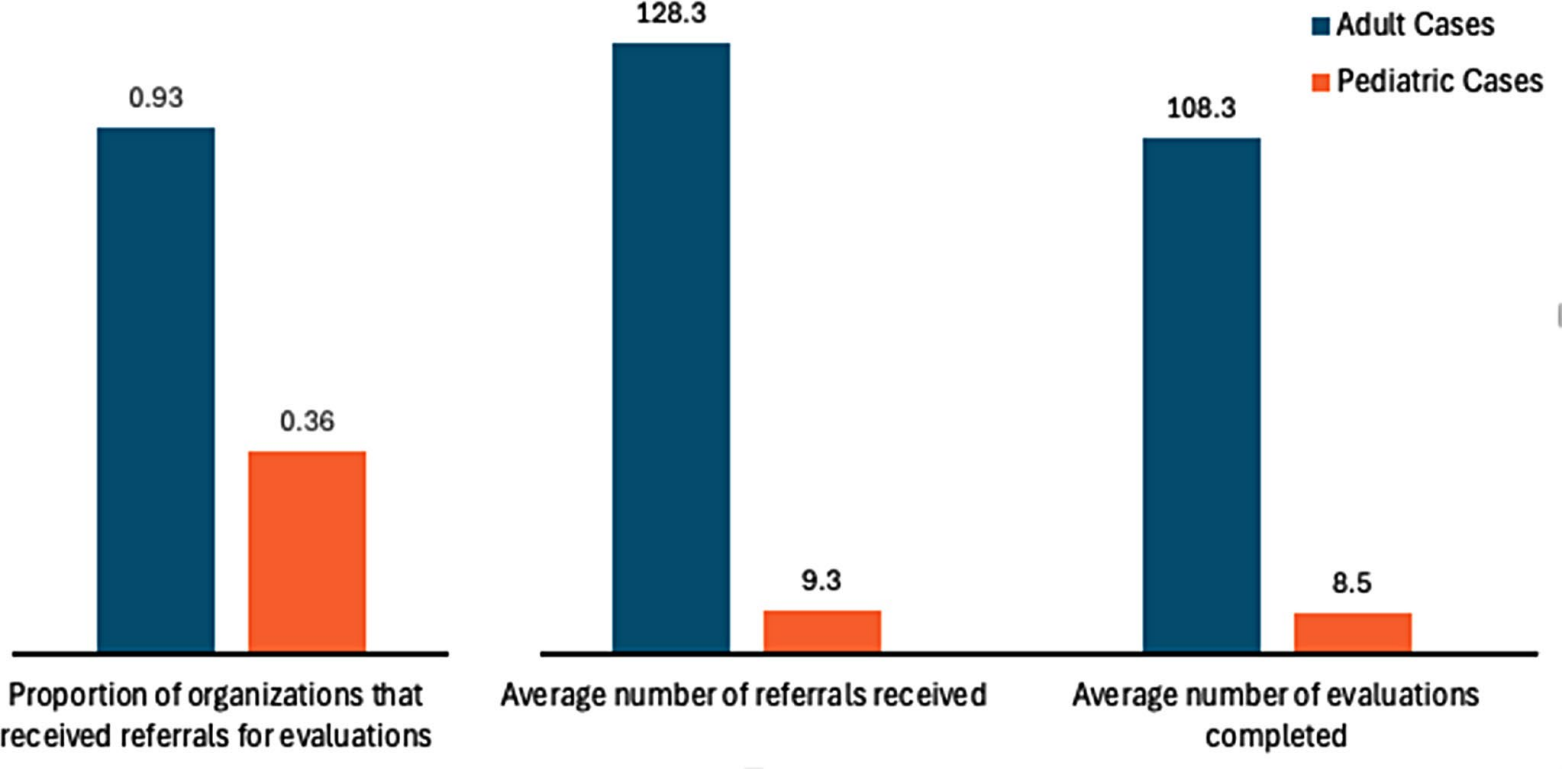
Comparing adult to pediatric operational capacity of student run asylum clinics

In contrast, 92.9% (*n* = 13) of the organizations received evaluation referrals for adult evaluations. Of the eleven organizations that provided complete survey responses, the mean number of adult evaluation referrals received in the past three years was 73.3 (SD = 90.8, range = 1–317) and the mean number of adult evaluations completed in the past three years was 55.5 (SD = 71.8, range = 0–245). For the four organizations that specified the number of pediatric forensic evaluations completed, the mean number of adult evaluations referrals received was 128.3 (SD = 132.8, range = 20–317) and the mean number of adult evaluations completed was 108.3 (SD = 100.0, range = 20–245) (Fig. 1).

**Fig. 2:**
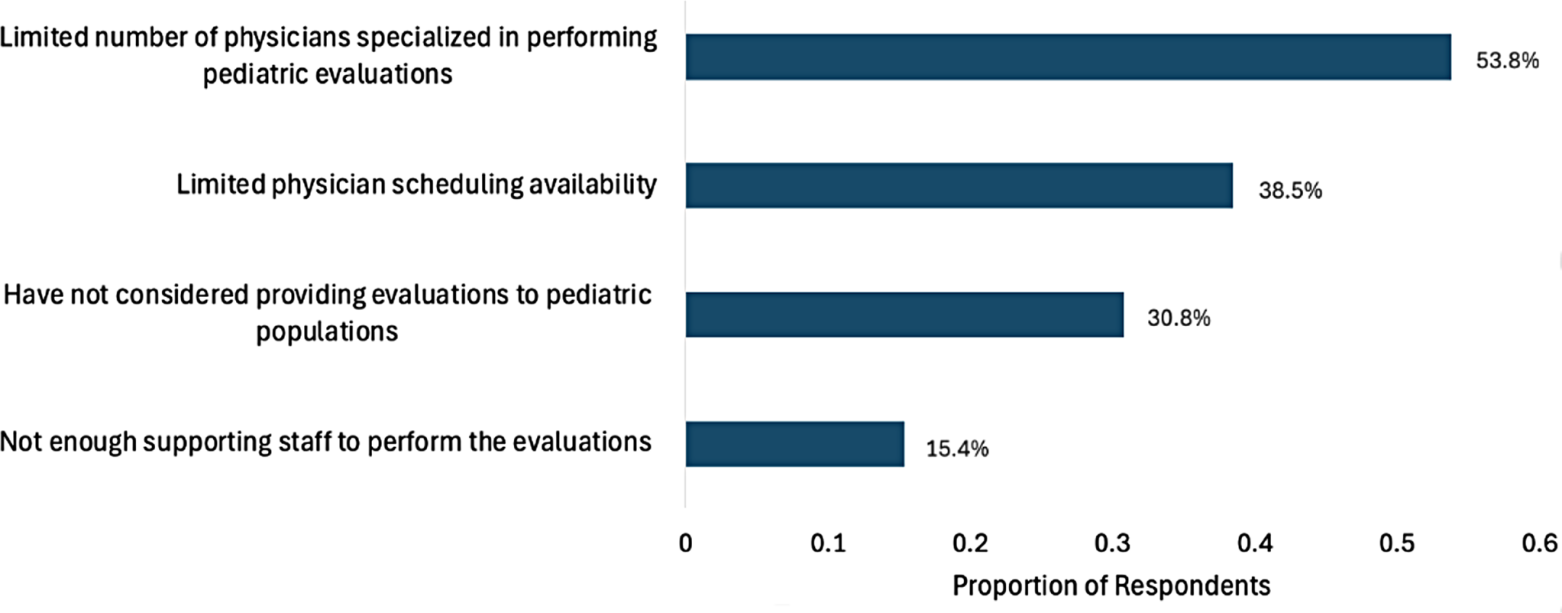
Barriers to providing forensic evaluations to pediatric populations

Regarding barriers that organizations encountered when considering or attempting to provide forensic evaluations for pediatric populations, thirteen organizations responded. 53.8% (*n* = 7) indicated that a limited number of physicians specialized in performing pediatric evaluations was a barrier. 38.5% (*n* = 5) indicated that limited physician scheduling availability was a barrier. 15.4% (*n* = 2) indicated that there was not enough supporting staff to perform the evaluations (i.e., source clients, arrange visits, arrange transportation, etc.). 30.8% (*n* = 4) indicated that they had not considered providing evaluations to pediatric populations (Fig. 2).

## Discussion

Our results of a sampling of SRACs show that the number of pediatric forensic evaluations performed is minimal relative to the number of adult forensic evaluations performed from 2021 to 2024. Furthermore, only one-third (35.7%) of organizations recognized a need for pediatric forensic evaluations in their area, despite the well-documented needs of pediatric asylum seekers in the United States.

Addressing barriers to performing pediatric forensic evaluations is a plausible way to increase the availability of this service. In our study, the most notable barrier to providing pediatric forensic evaluations was the limited number of physicians specialized or trained in performing pediatric evaluations. This finding is consistent with a prior survey study highlighting the scarcity of physicians who perform pediatric evaluations [13]. While several low-cost, well-established resources are available for providers to learn how to provide forensic medical evaluations to individuals seeking asylum, these resources typically focus more heavily on adult populations [16]. As such, one method to increase the number of providers with the specialized training needed to perform pediatric forensic medical and psychiatric evaluations is to create and disseminate standardized, low-cost training materials focused on pediatric populations. Training can also be integrated into elective educational curricula for providers that are in the process of completing training in the field of pediatrics. Of note, there are already a few pediatric training resources that can be further expanded upon or made more widely known [4, 16, 20, 21]. Broadly speaking, there are currently no national licensures available or required for providers interested in performing asylum evaluations, although general competencies have been defined and individual organizations that perform evaluations may have their own training requirements [8]. For instance, our organization requires completion of the modules available at no cost by the Asylum Medicine Training Initiative [22] before individuals can participate in medical and psychiatric evaluations.

The second most common indicated barrier to performing pediatric forensic evaluations was limited physician availability. While limited physician availability may be due in part due to the shortage of physicians with specialized training in this area, medical evaluations of this nature are usually done on a voluntary basis, which may further limit the pool of available providers [21]. Given that at least ten different provider types can perform pediatric forensic evaluations [4], one possibility to overcoming limited physician availability is to increase awareness of the need for these services and expand training programs to other types of providers such as nurse practitioners, physician assistant, and psychologists. This would increase the number of providers available to provide forensic evaluations to children and lessen the barrier of limited physician availability.

This study has several limitations. Notably, this study was not inclusive of all clinics due to a lack of contact information or response to request for participation. As such, the results did not have enough power to undergo comparison testing and are also not broadly generalizable. Furthermore, the organizations surveyed had substantial variability in the extent of their operations; some organizations served only a few clients, while others served hundreds. This difference led to considerable variability in our data and reflected the diverse environments in which forensic evaluations are conducted.

Our study suggests an unmet need for forensic evaluations among asylum-seeking children in the United States given the persistent number of children seeking asylum and survey results showing some organizations noting a need for pediatric evaluations, while others remain unaware of the need. While further knowledge of the scope of practice of student run asylum clinics should be gathered through continued data collection, our results highlight a broader need in the community. Given the documented benefits of forensic evaluations in supporting legal cases, it is of great pertinence to expand forensic medical evaluation resources for both accompanied and unaccompanied children seeking asylum in the United States. To further understand the amount of forensic medical and psychological evaluations being completed for children beyond SRACs, additional organizations that perform these evaluations, such as humanitarian organizations, refugee clinics, and private practices should also be surveyed regarding the extent of their services for children.

### New Contribution to the Literature

To our knowledge, this is the first ever study to survey student-run asylum clinics (SRACs) on the scope of their pediatric practice. SRACs are an increasingly common avenue for individuals seeking asylum to receive forensic medical evaluations and increase the likelihood of being granted asylum. Given this, SRACs can feasibly play a role in increasing accessibility to service that is scarce and needed by both accompanied and unaccompanied refugee children.

## Data Availability

Data relevant to the findings of this manuscript are available from the corresponding author upon request.
